# Polysaccharides from Radix Hedysari exhibited preventive effects against DSS-induced ulcerative colitis in C57BL/6 mice

**DOI:** 10.3389/fimmu.2026.1944924

**Published:** 2026-10-02

**Authors:** Lujie Ma, Yu Han, Xueqin Zhao, Yixiu Niu, Yuxin Xie, Yating Pan, Yaxin Zheng, Zhiyuan Xue

**Affiliations:** Key Laboratory of Structure-Specific Small Molecule Drugs at Chengdu Medical College of Sichuan Province, School of Pharmacy, Chengdu Medical College, Chengdu, China

**Keywords:** 16S rRNA sequencing, Hedysarum, inflammation, intestinal microbiota, polysaccharides, ulcerative colitis

## Abstract

Hedysarum polysaccharide (HPS), the major bioactive constituent of Radix Hedysari, possesses anti-inflammatory activity; however, few studies have explored its application for ulcerative colitis (UC). In this study, HPS-80 was extracted using combined complex-enzyme hydrolysis and ultrasonication. We further evaluated its preventive effects and underlying mechanisms in a dextran sulfate sodium (DSS)-induced UC model in C57BL/6 mice. Results showed HPS-80 significantly improved DSS-induced UC symptoms (weight loss, hematochezia, colon shortening) and reduced Disease Activity Index (DAI). Mechanistically, it inhibited pro-inflammatory factors Tumor Necrosis Factor-alpha(TNF-α),Interleukin-6, (IL-6), alleviated oxidative stress, upregulated tight junction proteins occludin, claudin-1(ZO-1), and modulated intestinal microbiota (suppressed harmful bacteria like Bacteroidota, enriched beneficial ones like Firmicutes and Akkermansia). Conclusion: HPS-80 exerts protective effects against UC via anti-inflammatory, antioxidant and gut-microbiota-regulating capacities. It is a promising candidate agent for UC prevention.

## 1 Introduction

UC is a chronic inflammatory bowel disease (IBD) affecting millions of people worldwide. It is defined by relapsing-remitting mucosal inflammation localized to the colon. Although substantial research progress has been achieved, the exact pathogenesis of UC has not been completely clarified. UC pathogenesis is linked to multiple risk factors, including diet, environmental exposure, genetic susceptibility, epithelial barrier impairment, dysregulated immune responses, and alterations in intestinal microbiota. A hallmark of UC is its recurrent nature (1) Most patients with UC exhibit symptoms including rectal bleeding, frequent bowel movements, abdominal pain, diarrhea, weight loss, and other related manifestations (2). Over the past decade, the global incidence of UC has been on the rise (3). Currently, therapeutic options include aminosalicylates, corticosteroids, immunosuppressive agents, and biological therapies. However, these treatments have inherent limitations and do not offer a complete cure for UC. They primarily function by reducing inflammation of the intestinal mucosal barrier and extending the remission period of the disease. Certain therapeutic drugs are associated with side effects, including drug resistance and adverse reactions (4). Despite ongoing efforts by numerous researchers to elucidate the pathogenesis of ulcerative colitis (UC) and develop innovative therapies, UC remains a challenging condition to manage. Consequently, there is an increasing interest in the development of cost-effective, efficacious, and low-toxicity agents or functional foods for UC management.

Recent studies have demonstrated that natural plant polysaccharides exhibit a range of physiological activities, including anti-inflammatory, antioxidant, antitumor, hypoglycemic, immune-regulatory, and gut microbiota-modulating effects. Due to their low toxicity, high safety profile, and promising therapeutic efficacy, these polysaccharides hold significant research value and development potential in modern medicine (5, 6). In the context of UC treatment, there is a growing focus among researchers on natural plant polysaccharides. For instance, Cui et al. identified a homogeneous polysaccharide, SP2-1, from Scutellaria baicalensis Georgi, which was shown to mitigate weight loss in DSS-induced UC mice, restore the colonic barrier, suppress inflammatory cytokine levels, and modulate the intestinal microbiota (7). Huo et al. demonstrated that Astragalus polysaccharide APS1 possesses prebiotic properties, which can ameliorate intestinal flora imbalance induced by DSS by promoting the proliferation of beneficial microbes and inhibiting the growth of potential pathogens (8). Similarly, Li et al. reported that Lycium barbarum polysaccharide (LBP) can mitigate ulcerative colitis in mice by decreasing inflammatory cytokines and enhancing the expression of tight junction proteins, while exhibiting strong prebiotic activity and modulating the intestinal microbiota (9).

Radix Hedysari, derived from the dried root of Hedysarum polybotrys Hand.-Mazz., has been the subject of recent studies focusing on HPS for their potential in mucosal repair and inflammation modulation. Yang et al. introduced an innovative extraction technique that combines enzymatic hydrolysis with ultrasonication to isolate three novel HPS fractions: HPS-MC, HPS-MC (50%), and HPS-MC (80%) (10). Building on this foundation, Xue et al. demonstrated that HPS-80 (HPS-MC 80%) significantly ameliorated acetic acid-induced gastric ulcers in rats and protected GES-1 gastric epithelial cells from H_2_O_2_-induced oxidative damage *in vitro*, thereby confirming its efficacy in repairing the mucosal barrier (11, 12). In addition, the intestine serves not only as a host for microorganisms but also houses a complex and extensive intestinal mucosal immune system (13). Disruption of the intestinal flora can compromise the integrity of the intestinal mucosa and epithelial cells, leading to inflammation and potentially progressing to cancer (14). Most plant polysaccharides are subject to fermentation by intestinal microorganisms, resulting in the production of various metabolites. This interaction facilitates the prebiotic activity of plant polysaccharides, which enhances the relative abundance of beneficial bacteria while reducing harmful bacteria within the intestine. Consequently, this process mitigates intestinal inflammation, augments microbial diversity, promotes mucosal repair, and restores a balanced and healthy state of the intestinal microflora (15). The physiological activity of Radix Hedysari polysaccharide (HPS-80) parallels that of these plant polysaccharides, suggesting its potential preventive efficacy against UC.

DSS is known to induce damage to intestinal epithelial cells, thereby creating a model that closely resembles human UC in both morphology and symptomatology (16). The DSS-induced model is recognized for its simplicity, stability, and maturity, making it the predominant method for UC modeling in mice. Consequently, this study employed the administration of a DSS-containing aqueous solution to mice via free drinking to induce ulcerative colitis. We will systematically observe and document the macroscopic characteristics of the UC-affected mice and conduct Disease Activity Index (DAI) scoring. The study will further analyze the mucosal repair capacity of the UC mice through histopathological examination, and intestinal permeability will be quantitatively assessed using immunohistochemistry (IHC). Additionally, inflammatory cytokines and oxidative stress markers will be measured to assess the anti-inflammatory and antioxidant properties of HPS-80. The composition of the intestinal microbiota will be analyzed using 16S rRNA high-throughput sequencing. This comprehensive evaluation aims to elucidate the protective effects of HPS-80 on UC in mice from multiple perspectives, thereby contributing novel insights and methodologies to the scientific research on the prevention and treatment of UC.

## 2 Materials and methods

### 2.1 Materials and equipment

HPS-80 was offered by Lanzhou University. DSS (Cat. No. D808272-100g, Lot: C17143789) was brought from Shanghai Macklin Biochemical Technology Co., Ltd. (Shanghai, China). 5-Ami-nosalicylic Acid (5-ASA) were purchased from Heilongjiang Timehome Pharmaceutical Co., Ltd. (Heilongjiang, China). The primary antibody (Abcam, catalog number: ab216347) was purchased from Wuhan Boster Biological Technology., Ltd., (Wuhan China),and the secondary antibody (Abcam) was purchased from BaiAo Biotechnology Co., Ltd., (Hubei, China).Sodium carboxymethyl cellulose (CMC-Na). Total protein quantitative kit (Coomassie brilliant blue method, A045-2-2), Malondialdehyde (MDA, A003-1-2) assay kit (TBA method), Total antioxidant capacity (T-AOC, A015-2-1) assay kit (ABTS method) Microplate technique, Total superoxide dismutase (T-SOD, A001-1-2) assay kit (hydroxylamine method)kits were provided by Nanjing Jiancheng Bioengineering Institute (Nanjing, China). Normal goat serum(AR1009) were offered from Wuhan Boster Bioengineering Co., Ltd.(Wuhan, China).

### 2.2 Preparation of Radix Hedysari polysaccharide

The ground dry roots of Radix Hedysari powder (10 kg) were smashed, sieved through an 80-mesh sieve, and pre-extracted three times with acetic ether and 95% ethanol (1:8,1:6,1:6, w/v) at 100 °C for 2 h each time, respectively. And 5675.1 g of the degreased powder was obtained after drying in an oven at 50 °C. Next, the defatted powder was weighed and socked with distilled water (1:30, w/v) at 50 °C for 2 h, in the process, the pH value of supernatant was adjusted to 5.0-5.4 with citric acid buffer (2%, w/w), and the relevant 2% complex enzyme (papain: cellulose =1:1, w/w) preparations were added. After enzymatic hydrolysis, put it in an ultrasonic cleaner (KQ-3200DE, Kunshan ultrasonic instrument Co., Ltd., China)for extracting in a water bath at 60 °C and ultrasound power 105 W for three cycles at 1.5 h each. It’s important to note that the latter two ultrasound treatments add water(1:20,1:10, w/v). After that, the combined aqueous extracts were concentrated to 1/10 of the initial volume with a rotary vacuum evaporator(SHZ-D(III). Next, 95% ethanol was added to the concentrated supernatant with continuous stirring to achieve a final concentration of 80% ethanol and this solution was kept at 4°C for 24 h. Then, the crude polysaccharide from Radix Hedysari, named HPS were collected by centrifugation (6000 rpm for 10 min) (AllegraTM64R centrifuge, Beckman Coulter Co., USA). and dried under reduced pressure. The resulting 615.0 g HPS was dissolved in distilled water (1:20, w/v) by incubation in a water bath at 60°C. A step-ethanol precipitation was done according to the method of with appropriate modifications (17). 95% ethanol was added to the HPS supernatant with continuous stirring to achieve a final concentration of 50% ethanol and this solution was kept at 4°C for 24 h. The precipitated polysaccharides (HPS-50) were then collected by centrifugation as described above. According to this method, HPS-80 was collected. The two HPS fractions were dried under lyophilization.

As previously reported, the total carbohydrate content of HPS-80 determined by the phenol-sulfuric acid method was 92.25 ± 2.44 %. Its protein content was 3.28 ± 0.60 % determined by Coomassie brilliant blue assay, and uronic acid content was 1.93 ± 0.30 % measured by sulfuric acid-carbazole assay. HPSEC-MALS analysis showed its weight-average molecular weight (Mw) was 6.49 × 10³ Da with a polydispersity index of 1.30, indicating a relatively narrow molecular-weight distribution (10).

### 2.3 Animals and experimental design

6–8 weeks old male C57BL/6 mice (18-22g) were purchased from Chengdu Dossy Experimental Animals Co., Ltd (Chengdu, China). Before the start of the experiment, all animals were housed in a properly maintained room with free access to food and drinking water, all mice were in a controlled environment (20 ± 5°C, RH 60 ± 5%) and under a 12-hour light-dark cycle for 5 days. For animal grouping, all mice were numbered and allocated to experimental groups using a random-number table to minimize selection bias. All scoring assessments, including the Disease Activity Index (DAI), histopathological lesion scoring, and IHC protein quantification, were performed under single-blind conditions. Evaluators remained unaware of group assignments for individual mouse samples throughout the scoring procedure. Each animal procedure is conducted in accordance with the guidelines of the Animal Care and Ethics Committee of Chengdu Medical College (Approved by Animal Protection and Use Ethics Committee of Chengdu Medical College (Certificate No. SYXK (Sichuan) 2020-0196).

Forty-nine male C57BL/6 mice (18–22 g) were divided into 6 groups: Control group (C), DSS group (M), Positive group (5-ASA, P), Radix Hedysari polysaccharide low dose group (HPS-80-L), Radix Hedysari polysaccharide medium dose group (HPS-80-M), Radix Hedysari polysaccharide high dose group (HPS-80-H), with 8 mice in each group (9 mice in Control group).The initial scheme set 8 animals for each group (total of 48 required mice). One surplus mouse was obtained after grouping; to abide by animal ethical regulations against redundant euthanasia, this extra mouse was allocated to the Control group, leading to 9 mice in Control group and 8 mice in each of the other five groups. Except for the Control group, the mice in the other groups were free to drink 3% (w/v)DSS distilled water for 7 consecutive days, and the DSS distilled water was changed every 2 days. At the same time, HPS-80 -L,M,H(100,200,400 mg/kg/d) and 5-ASA (200 mg/kg/d) were mixed with 0.5% CMC-Na as a dissolvant to prepare a suspension for mice. The mice were given intragastric administration at a regular time every day. The Control group and the DSS group were intragastrically administered with 0.5% CMC-Na at 0.1 ml/10g. The drinking water of each group was observed by measuring the weight of the remaining water. All mice were sacrificed by cervical dislocation on the 8th day after administration.

### 2.4 Assessment of disease activity index based on body weight, stool characteristics and rectal bleeding

During the experiment, indicators such as body weight, vitality, hair luster, fecal morphology and stool blood of mice in each group were observed and recorded at a fixed time every day to evaluate DAI score. The DAI score was combined with the percentage of patient ‘s weight loss, stool viscosity and stool bleeding to make a comprehensive score, in order to observe the macroscopic preventive intervention between different groups.

### 2.5 Colon length and organ index

On the 8th day after administration, the mice were sacrificed by cervical dislocation. The colon tissue of 5 cm from the distal end of the anal orifice was immediately removed by laparotomy, photographed and recorded, and the wet weight was weighed on a clean filter paper. Immediately, the spleen and liver were taken out and weighed on a clean filter paper to calculate the organ index. (Organ index = organ weight/mouse body weight).

### 2.6 Cytokines and measurement of oxidative stress

Before the mice were sacrificed, the blood of the mice was obtained by eyeball extraction, and the serum was obtained by centrifugation (2500 r/min, 15 min)(D-37520,Thermo Electron Corporation, China) and immediately frozen in a refrigerator at-80°C.

The colon of the mice was removed quickly, and half of it was cut and immediately added with 9 times the volume of cold normal saline according to the weight (g): volume (mL) = 1: 9. The high-throughput tissue grinder (60 Hz, 60 s) (SCIENTZ-24, Ningbo Xinzhi Biotechnology Co., Ltd. China) was used to make 10% homogenate under ice water dissolution conditions. The supernatant was centrifuged (1500 r/min, 5 min) and stored in a refrigerator at−80°C. Total protein quantification, ROS,T-SOD, T-AOC activities, and MDA content were measured with kits. All experimental operations and data processing were carried out according to the manufacturer ‘s instructions. The T-SOD assay kit (Cat. No. A001-1), T-AOC assay kit (Cat. No. A015-2-1), MDA assay kit (Cat. No. A003-1), and GSH assay kit (Cat. No. A006-2-1) were obtained from Nanjing Jiancheng Bioengineering Institute. ELISA kits for TNF-α (Cat. No. EM0183), IL-6 (Cat. No. EM0121), IL-1β (Cat. No. EM010), and IFN-γ (Cat. No. EM0093) were purchased from Wuhan FineTest Co., Ltd. All measurements were performed strictly following the manufacturer’s instructions.

### 2.7 Colon histopathology

The colon tissues of 3 mice in each group were fixed with 4% paraformaldehyde and embedded in paraffin, sliced into 4 μm sections and stained with Hematoxylin-Eosin (H&E), per-iodic acid-Schiff (PAS) and Masson to evaluate the intestinal morphology of mice. Only 3 mice from each group were selected for immunohistochemical staining and detection in this study. Colonic injury was assessed using the validated Rachmilewitz scoring system. Five parameters including depth of ulcer, extent of ulcer, presence of inflammation, extent of inflammation and location of fibrosis were evaluated, each scored from 0 to 4. The total histopathological score was the sum of five sub-scores, with a maximum total score of 20. All paraffin sections were randomly coded for blind evaluation by two independent researchers. Discrepant scores were resolved by joint re-examination.

### 2.8 Immunohistochemistry

The sections were dewaxed in the environmentally friendly dewaxing agent (1–3) for 10 minutes, and then subjected to anhydrous ethanol, 95% ethanol, and 75% ethanol for 5 minutes each. Wash with distilled water 3 times, then rinse. Then the antigen was repaired with Ethylenediaminetetraacetic Acid(EDTA) (pH 9.0), citric acid (pH 6.0) and microwave (EDTA or citric acid) (Hubei Baiaosi Biotechnology Co., Ltd., China) in turn. After three times of repair and cooling, the sections were taken out and light-shielded blocked with 3% H_2_O_2_ (Sinopharm Chemical Reagent Co., Ltd., China) at room temperature for 30 minutes, and then circled. Subsequently, 10% normal goat serum (Boster Biological Technology Co., Ltd., Wuhan, China) was added dropwise, followed by incubation at room temperature for 30 minutes. The primary antibody(Wuhan Boster Biological Technology., Ltd., China) was incubated overnight at 4°C, and the secondary antibody(Hubei Baiaosi Biotechnology Co., Ltd., China) was incubated at 37°C for 45 minutes. Colored with DAB(Hubei Baiaosi Biotechnology Co., Ltd., China), hematoxylin reverse staining.

### 2.9 16S rRNA high-throughput sequencing was used to analyze the intestinal flora

Fecal samples were collected from four groups (Control, DSS, Positive, HPS-80-H) for 16S rRNA high-throughput sequencing. Paired fresh feces were harvested from each mouse at two time points: before DSS modeling and intragastric administration (pre-treatment), and on the 8th day after continuous treatment (post-treatment). All collected fecal samples were immediately transferred into sterile cryogenic tubes and stored at −80 °C until genomic DNA extraction. Each group contained 6 independent biological replicates, and all qualified fecal samples passing DNA extraction and sequencing quality control were included in the downstream microbial analysis (18, 19). The pre-treatment and post-treatment fecal samples from the identical mouse were matched paired samples to evaluate the dynamic changes of intestinal flora within individuals after intervention. Finally, all of them were sent to Beijing Biomarker Technologies Co., Ltd.(Beijing, China) for 16S rRNA high-throughput sequencing. Based on the Illumina NovaSeq sequencing platform, a small fragment library was constructed by Paired-End sequencing. The species composition of the samples can be revealed by Reads splicing filtering, clustering or denoising, and species annotation and abundance analysis. Further analysis of alpha diversity, beta diversity, significance analysis of inter-group differences, correlation and association analysis, functional prediction analysis, etc., can explore the differences between samples.

Based on our preliminary pharmacodynamic experiments, the therapeutic efficacy of HPS-80 against DSS-induced UC increased in a dose-dependent manner, and the HPS-80-H group displayed the most prominent protective effects on inflammatory response, intestinal mucosal barrier and oxidative stress. Therefore, prior to the formal high-throughput sequencing experiment, we preset the HPS-80-H group as the representative intervention group to investigate the modulatory effect of HPS-80 on intestinal microbiota, while the low- and medium-dose groups were not subjected to 16S rRNA sequencing in this study.

### 2.10 Statistical analysis

Statistical analysis was performed using SPSS 27.0 and QIIME 2 software. Prior to significance testing, normality of distribution and homogeneity of variance were assessed. One-way analysis of variance (ANOVA), α diversity analysis, β diversity analysis, significance analysis of differences between groups, correlation and association analysis, and functional prediction analysis were used to evaluate the differences between groups. *P* < 0.05 was considered statistically significant.

## 3 Results and discussion

### 3.1 Hedysari polysaccharide can reduce DSS-induced ulcerative colitis in mice

Observing the signs and symptoms in mice is crucial for the diagnosis of UC, which includes monitoring changes in body weight, colon length, fecal characteristics, and organ indices (20) During the experiment, mice in the Control group exhibited glossy fur, robust appetite, high levels of activity, and normal fecal morphology. In contrast, after three days of consuming a 3% DSS solution, mice in the DSS group demonstrated significant weight loss, reduced appetite, lethargy, loose stools with bleeding, among other symptoms. These observations confirm that a 3% DSS solution effectively induces UC in mice, with weight loss being a prominent macroscopic symptom of the condition (**Figures 1A, B**).

**Figure 1:**
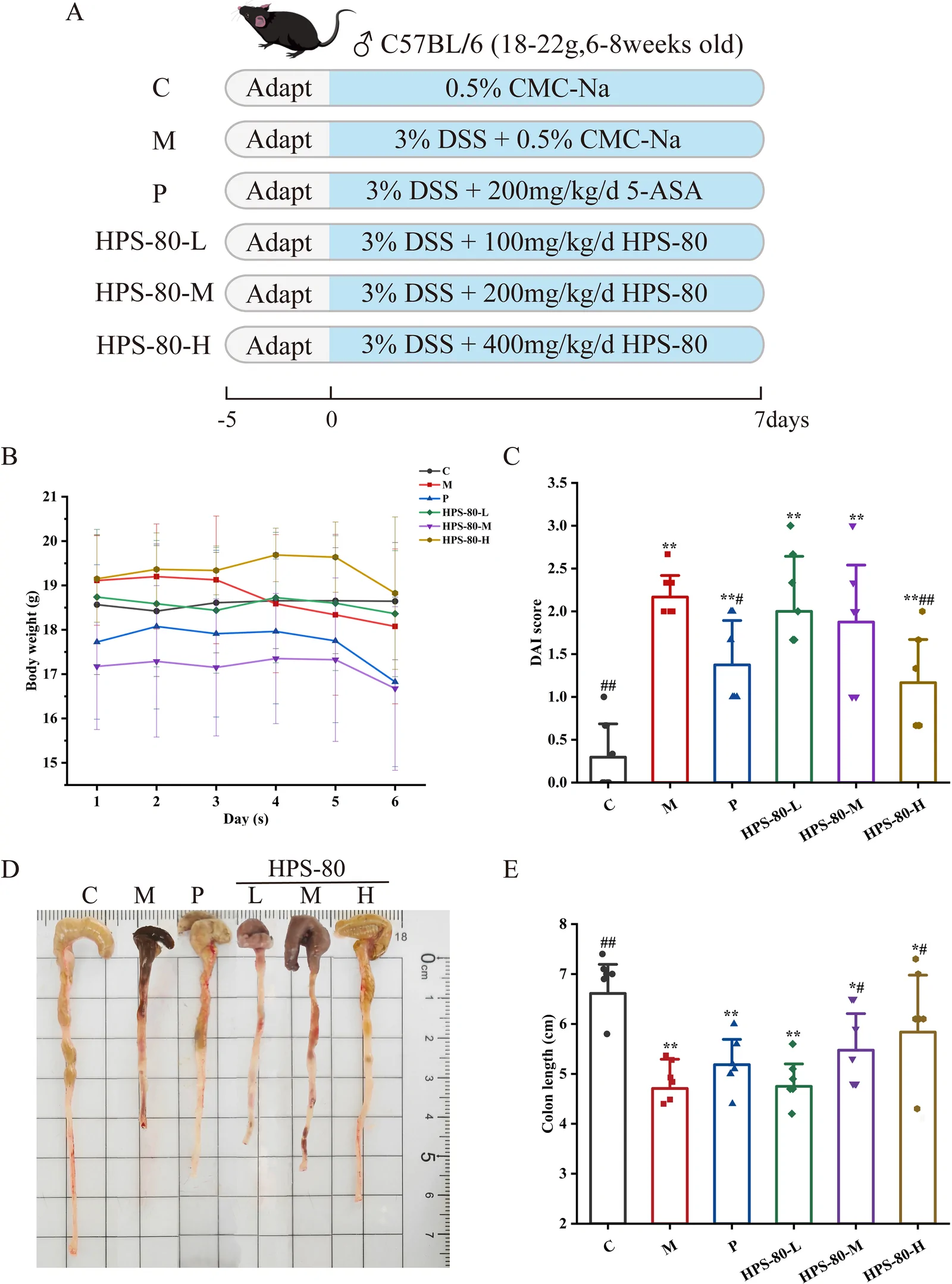
HPS-80 can alleviate DSS-induced ulcerative colitis in mice (n = 6). **(A)** Schematic diagram of animal experiment design. **(B)** Effects of HPS-80 on body weight. **(C)** DAI score **(D)** Colonic length diagram. **(E)** Colon length. Group C represents the Control group. Group M represents the DSS group. Group P represents the Positive group. Group HPS-80-L, HPS-80-M, HPS-80-H represent the low, medium and high dose groups of HPS-80, respectively. Data are expressed as mean ± SD (n=6). Compared with the control group, *P < 0.05; **P < 0.001; Compared with the DSS group, ^#^*P* < 0.05; ^##^*P* < 0.001.

While the body weight of the Control group showed a gradual increase, the body weight of the DSS group began to significantly decline three days post-DSS administration. Notably, treatment with HPS-80 and the positive control drug 5-ASA alleviated the symptoms of weight loss in the HPS-80 and Positive groups, respectively, when compared to the DSS group (**Figure 1B**).

The DAI score serves as a critical metric for assessing the severity of UC, encompassing the cumulative evaluation of body weight changes, fecal consistency, and fecal bleeding in murine models. A higher DAI score indicates a more severe manifestation of ulcerative colitis. The study results demonstrated that the DAI score in the DSS group was significantly elevated compared to the Control group (*P* < 0.001). Following the administration of HPS-80 and the positive control drug 5-ASA, a reduction in DAI scores was observed, with the HPS-80-H group and the Positive group showing significant decreases (*P* < 0.001) (**Figure 1C**).

Colonic shortening and splenomegaly are also critical parameters for evaluating UC severity (21). In comparison to the Control group (6.61 ± 0.58 cm), the colon length in mice subjected to DSS treatment was significantly reduced (4.71 ± 0.59 cm, *P* < 0.001), whereas treatment with HPS-80 and 5-ASA markedly ameliorated these symptoms. Specifically, the HPS-80-M (5.41 ± 2.04 cm) and HPS-80-H (5.84 ± 1.14 cm) groups exhibited significant preventive effects (*P* < 0.05), and the colon-length-improving effect showed a numerical tendency higher than that of the 5-ASA-treated group (**Figures 1D, E**), surpassing the efficacy of 5-ASA (**Figures 1D, E**). The spleen, a vital immune organ, frequently exhibits splenomegaly in UC patients (22, 23).In comparison to the Control group (2.77 ± 0.17 g/kg), the spleen weight index of the DSS group (3.47 ± 0.21 g/kg) showed a significant increase; however, this effect was reversed in the HPS-80 group (see **Supplementary Table S1**).

From a broader perspective, the preventive efficacy of HPS-80 on UC in mice was evident. HPS-80 was found to mitigate symptoms such as weight loss, colonic shortening, and splenomegaly, decrease the DAI score, significantly attenuate DSS-induced ulcerative colitis in mice, and exert both alleviating and protective effects on the condition.

### 3.2 Effect of Radix Hedysari polysaccharide on inflammatory factors in mice with ulcerative colitis

In this research, serum levels of inflammatory markers (TNF-α, IL-6, IL-1β, and IFN-γ) were assessed using an Enzyme-linked Immunosorbent Assay(ELISA) kit. Following treatment with 3% DSS, data indicated that HPS-80 significantly reduced serum inflammatory factor levels, implying its potential to mitigate DSS-induced inflammation. Research indicates that IL-6, IL-1β, TNF-α, and IFN-γ levels are elevated in the intestinal mucosa of UC patients, contributing to inflammation and worsening mucosal damage. TNF-α and IL-6, for instance, are crucial in UC progression, working together to enhance inflammation (24). ELISA findings revealed that DSS treatment markedly raised IL-6 and TNF-α serum levels in mice compared to the Control group (*P* < 0.001), Consistent with these cytokine changes, DSS-treated mice displayed typical UC clinical manifestations: body-weight loss, loose stools, and hematochezia. The DSS group also had higher DAI scores, shortened colons, and elevated histopathological semi-quantitative scores. Together, these quantitative and semi-quantitative indices confirmed successful establishment of the DSS-induced UC mouse model. The findings indicate that HPS-80 treatment significantly attenuated the expression of inflammatory factors (*P* < 0.001), with IL-6 and TNF-α levels decreasing in a dose-dependent manner with increasing HPS-80 concentrations (**Figures 2a1, a2**). Notably, the TNF-α level in the HPS-80-H group was reduced by 33%, showing a numerically larger reduction compared with the 31% decrease observed in the positive-control group, though no direct statistical comparison was performed between these two groups (see **Supplementary Table S2**). These results suggest that HPS-80 may ameliorate the inflammatory response by inhibiting the production of pro-inflammatory factors in DSS-induced ulcerative colitis.

**Figure 2:**
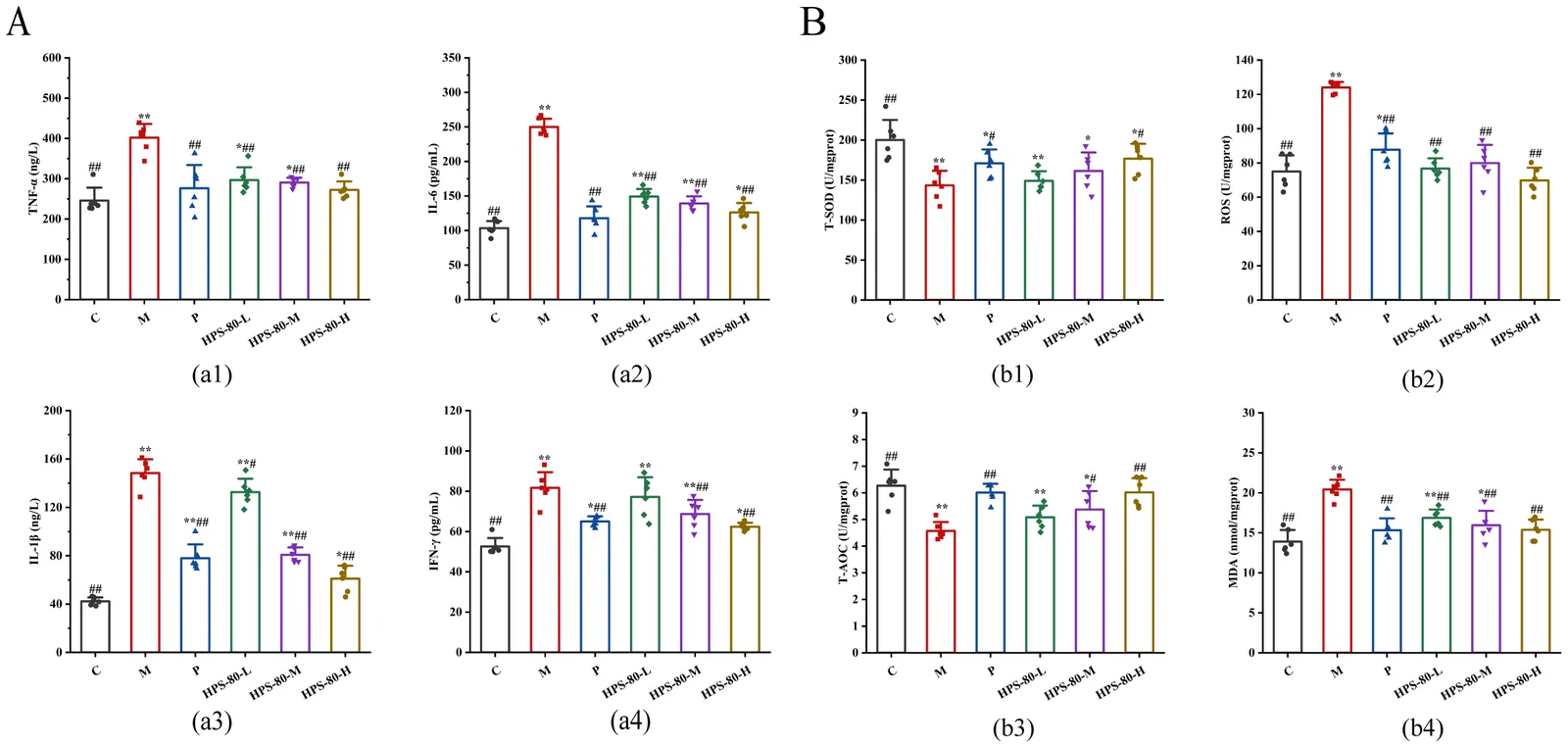
Effects of HPS-80 on inflammatory factors in mice with ulcerative colitis and HPS-80 can reduce the level of oxidative stress in mice. (n=6). **(a1)** TNF-α. **(a2)** IL-6. **(a3)** IL-1β. **(a4)** IFN-γ. **(b1)** T-SOD. **(b2)** ROS. **(b3)** T-AOC. **(b4)** MDA. Values are displayed as average ± SD. Compared with the control group, *P < 0.05; **P < 0.001; compared with the DSS group, ^#^*P* < 0.05; ^##^*P* < 0.001.

Interleukin-1β (IL-1β), a prototypical pro-inflammatory cytokine, can induce the release of other inflammatory cytokines, such as IL-6 and TNF-α. These cytokines further activate neutrophilic granulocytes and macrophages, which engulf invading pathogens and release toxic oxygen and nitrogen free radicals, thereby exacerbating the inflammatory response. IL-1β is also a multifunctional cytokine that affects nearly all cell types, either independently or in synergy with other cytokines (25, 26).

The ELISA results indicated that, relative to the Control group, DSS induction led to a significant elevation in serum IL-1β levels in mice (*P* < 0.001), with an increase of 141% (see **Supplementary Table S2**). Conversely, IL-1β levels were significantly reduced following HPS-80 treatment (*P* < 0.001). Specifically, compared to the DSS group, IL-1β levels decreased by 11%, 46%, and 59% in the HPS-80-L, HPS-80-M, and HPS-80-H groups, respectively. The Positive group exhibited a 47% reduction, with the HPS-80-H group demonstrating the most pronounced effect (see **Supplementary Table S2**; **Figure 2a3**).

Interferon-γ (IFN-γ) is a crucial soluble dimeric cytokine and the sole member of the type II interferon family. IFN-γ exerts a significant pro-inflammatory effect by releasing various inflammatory mediators and cytokines, such as nitric oxide (NO), interleukin-1 (IL-1), and tumor necrosis factor-alpha (TNF-α), through the activation of immune cells, including macrophages, natural killer (NK) cells, and T cells. These substances can further exacerbate the inflammatory response (27).The ELISA results indicated that the level of IFN-γ in a murine model of ulcerative colitis induced by DSS was significantly elevated compared to the Control group (*P* < 0.001). Both the Positive group and the HPS-80 group exhibited significantly higher levels of IFN-γ (*P* < 0.001), with the HPS-80-H group demonstrating the most pronounced therapeutic effect (**Figure 2a4**). In comparison to the DSS group, the IFN-γ levels in the HPS-80 group were reduced, with a further decrease observed as the HPS-80 dose increased. Specifically, the IFN-γ level in the HPS-80-H group decreased by 24%, compared to a 20% reduction in the Positive group, indicating a superior preventive effect (see **Supplementary Table S2**).

These findings suggest that HPS-80 may ameliorate the inflammatory response by inhibiting the production of pro-inflammatory factors in DSS-induced ulcerative colitis, with the reduction in inflammatory factor levels correlating with increased HPS-80 dosage, thereby enhancing the anti-inflammatory effect.

### 3.3 Effect of Radix Hedysari polysaccharide on oxidative stress in mice with ulcerative colitis

DSS induces an abnormal immune response in animals by compromising colonic epithelial cells, impairing epithelial barrier function, and allowing inflammatory substances in the intestinal lumen to infiltrate the lamina propria and submucosa. Oxidative stress, characterized by an imbalance between pro-oxidant and antioxidant processes within the body, is intricately linked to the pathogenesis of UC. Research has demonstrated that chronic inflammatory responses can exacerbate oxidative stress, primarily through the generation of oxidants and free radicals, which subsequently inflict damage and toxicity on cellular components such as lipids and DNA (28) Furthermore, extensive literature indicates a significant association between UC treatment and oxidative stress modulation (29). Consequently, we assessed the antioxidant capacity of HPS *in vivo* by evaluating oxidative stress markers in murine colon tissue.

Simultaneously, the concentrations of malondialdehyde (MDA) and reactive oxygen species (ROS) exhibited a significant increase (*P <* 0.001), with both rising by 40.0% (see **Supplementary Table S3**). These findings imply a compromised antioxidant capacity in ulcerative colitis (UC) mice and indicate that the DSS model markedly induces oxidative stress. In comparison to the DSS group, the HPS-80 treatment groups demonstrated elevated activity levels of total superoxide dismutase (T-SOD) and total antioxidant capacity (T-AOC) (**Figures 2b1, b3**), while MDA and ROS levels were significantly reduced (*P <* 0.001) (**Figures 2b2, b4**). Detailed analysis revealed a dose-dependent relationship, with the HPS-80 high-dose group (HPS-80-H) exhibiting the most pronounced effects (*P <* 0.001), characterized by a 23.3% increase in T-SOD, a 33.1% increase in T-AOC, a 27.2% decrease in MDA, and a 43.7% decrease in ROS. No significant differences in oxidative stress and antioxidant capacity were observed between the HPS-80-H group and the Control group, as evidenced by an 11.6% decrease in T-SOD, a 3.0% decrease in T-AOC, a 7.0% increase in MDA, and a 6.3% increase in ROS (see **Supplementary Table S3**). These results suggest that HPS-80 exerts a substantial effect in ameliorating the oxidative stress condition in the colon.

In the current study, four pro-inflammatory cytokines (TNF-α, IL-6, IL-1β, and IFN-γ) were selected to evaluate systemic inflammatory status. According to authoritative reviews and classic animal experiments of DSS-induced colitis, these four cytokines are highly representative biomarkers for ulcerative colitis mouse models (18, 19). TNF-α and IL-1β act as early innate immune mediators to initiate acute intestinal inflammatory cascades. IL-6 serves as a central regulator bridging innate and adaptive immunity, which continuously mediates chronic mucosal inflammatory injury. As a typical Th1-type signature cytokine, IFN-γ further disrupts the integrity of the intestinal epithelial barrier.

These cytokines are well-documented signature markers of Th1-type immune responses that dominate DSS-induced colitis, reflecting systemic inflammatory activation in UC mouse models. TNF-α acts as an upstream initiating factor, which triggers the release of downstream IL-6 and IL-1β from macrophages and colonic epithelial cells; IL-1β further amplifies inflammatory cascades and promotes IFN-γ secretion from T-lymphocytes, while elevated IFN-γ in turn potentiates TNF-α-driven mucosal injury, forming a positive-feedback inflammatory loop in colonic lesions (30). Our results demonstrated that HPS-80 intervention simultaneously suppressed the elevation of all four cytokines in a dose-dependent manner, implying that HPS-80 may break this synergistic pro-inflammatory cascade rather than merely inhibiting a single cytokine.

Notably, HPS-80 demonstrates substantial antioxidant activity within colon tissue during DSS-induced ulcerative colitis treatment, offering a robust preventive foundation for the development of drugs or functional foods with potential anti-ulcerative colitis properties.

### 3.4 Histopathological changes of colon in mice

Histological alterations were assessed using H&E and PAS staining of colon tissue samples. In healthy mice, the colon tissue exhibited a random distribution of inflammatory cells and an intactepithelial architecture (31). The presence of well-formed glands and crypt structures was noted, which are capable of secreting mucus to establish a mucosal barrier, thereby protecting the epithelial cells. The experimental results indicated an absence of significant histological abnormalities in the Control group. In contrast, the colon architecture of the DSS group displayed disarray, crypt destruction, a marked reduction in goblet cell numbers, and increased infiltration of inflammatory cells into the intestinal mucosa and muscularis layer (**Figure 3A**).Consequently, the histopathological score was significantly elevated (see **Supplementary Table S4**).

**Figure 3:**
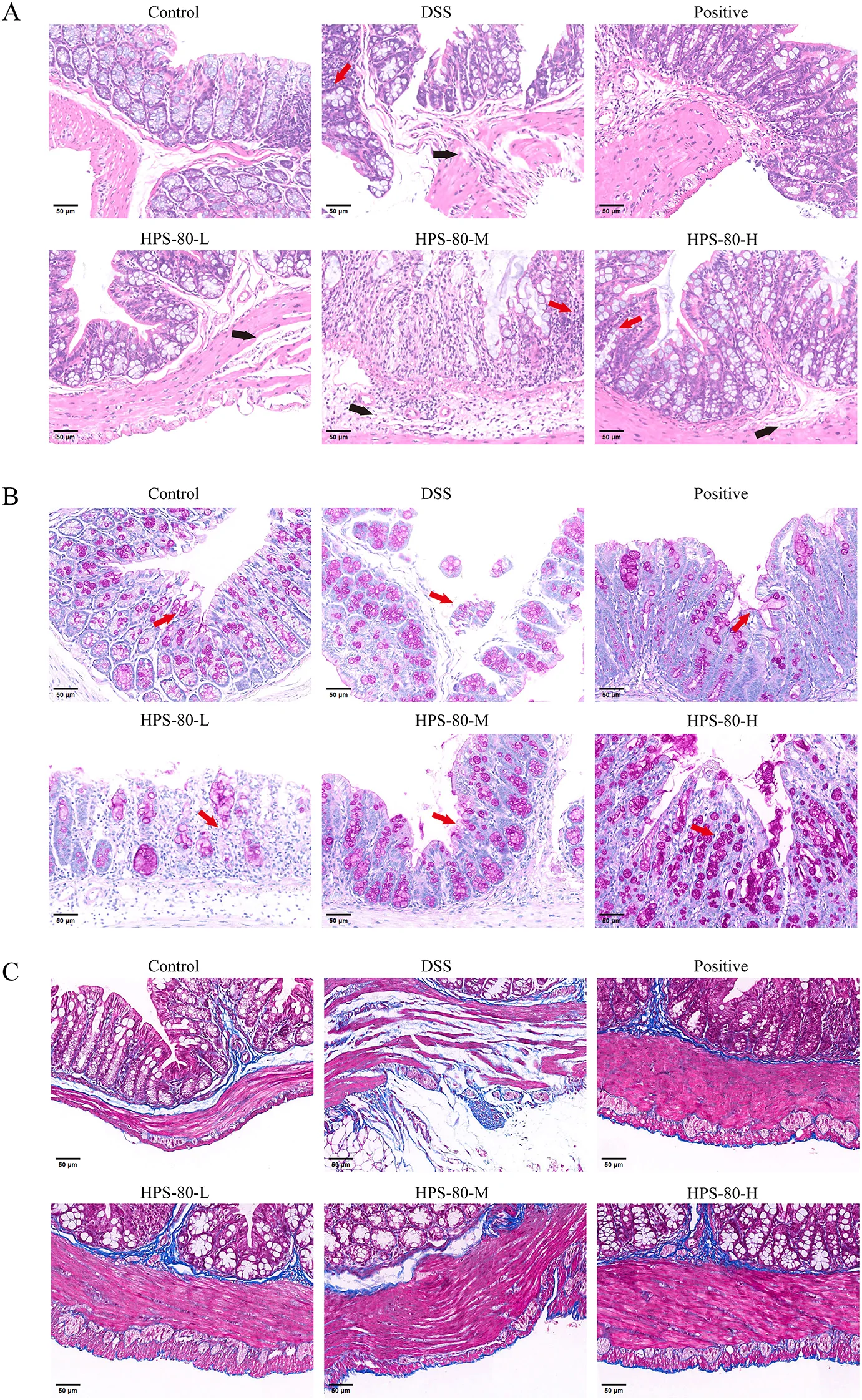
Hedysarum Polysaccharides can alleviate DSS-induced colon tissue injury in mice. **(A)** HE (scale bar, 50 μm). **(B)** PAS (scale bar, 50 μm) **(C)** Masson (scale bar, 50 μm). The red arrow points to the goblet cells and the black arrow points to the missing part of the mucosal layer.

In comparison to the DSS group, the Positive group and the HPS-80-H group exhibited reduced inflammatory cell infiltration, a clearer and more intact colon structure, restored crypt architecture, and an abundance of goblet cells, resulting in diminished inflammatory cell infiltration (32). PAS staining further revealed a pronounced staining of intestinal mucus within the crypt tissue of mice, indicating a substantial presence of intestinal mucus (**Figure 3B**). Masson staining analysis demonstrated severe fibrosis in the DSS group, whereas the distribution of collagen fibers in the mucosa and submucosa was less pronounced and more orderly in each HPS-80 dose group, indicating a relatively mild degree of fibrosis. These findings suggest that HPS-80 mitigates the degree of fibrosis in colon tissue by restoring the barrier function of the intestinal mucosa, enhancing mucus secretion and crypt structure, and increasing the number of goblet cells. Consequently, this leads to a reduction in the inflammatory response, with the anti-inflammatory effect becoming more pronounced at higher concentrations of HPS-80 (**Figure 3C**).

### 3.5 Immunohistochemistry

Comprehensive evidence of intestinal barrier restoration suggests that the integrity of both the mechanical and chemical components of the intestinal barrier serves as a crucial physical defense against pathogenic invasion. Research has demonstrated that yam polysaccharides significantly facilitate the repair of intestinal epithelial damage by upregulating the expression of tight junction proteins, including ZO-1 and Occludin, and by reducing levels of serum endotoxin (ET) and lipopolysaccharide binding protein (LBP). Polysaccharides contribute to the reconstruction of barrier function through a multidimensional synergistic approach, encompassing mechanical repair, antioxidative activity, and enhancement of the mucus layer (33).Mucosal damage can lead to increased intestinal permeability, trigger various inflammatory responses, and compromise the intestinal mechanical barrier. To assess the integrity of the intestinal barrier (34), IHC was employed to detect the expression of three key proteins: ZO-1, occludin, and claudin-1. These proteins are predominantly expressed in the colonic mucosal epithelial cells of mice and play a crucial role in maintaining intestinal integrity and permeability. In comparison to the Control group, the expression levels of these proteins were significantly reduced in the DSS group. However, their expression was restored to varying extents in the Positive group and the HPS-80 treatment groups. Notably, the HPS-80-H group demonstrated superior efficacy compared to the other two treatment groups, with significantly better outcomes than the DSS group (**Figures 4A–D**). These findings suggest that HPS-80 has the potential to mitigate DSS-induced colon tissue damage in mice, protect epithelial cells, and partially preserve the integrity of the intestinal mucosa.

**Figure 4:**
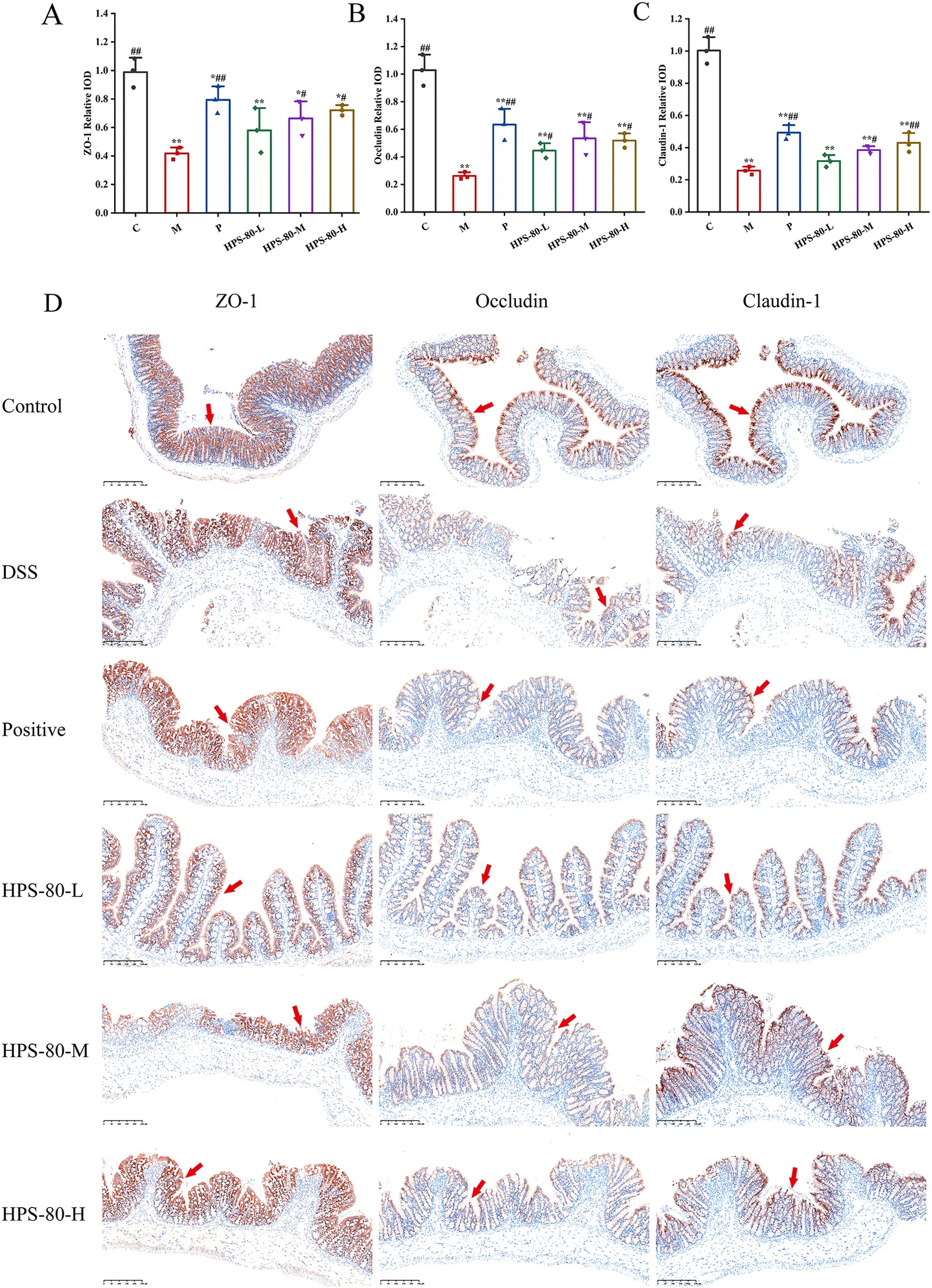
HPS-80 can alleviate DSS-induced colon tissue injury in mice. **(A)** ZO-1 relative value. **(B)** Occludin relative value. **(C)** Claudin-1 relative. **(D)** IHC (scale bar, 250 μm).The red arrow points to the protein expression situation; Group C stands for Control group; Group M stands for the DSS group. Group P stands for the Positive group; HPS-80-L, HPS-80-M and HPS-80-H stands for the low, medium and high dose groups of Radix Hedysari Polysaccharides. Data are expressed as mean ± SD (n=3). Compared with the control group, *P < 0.05; **P < 0.001; Compared with the DSS group, ^#^*P* < 0.05; ^##^*P* < 0.001.

### 3.6 Effects of Radix Hedysari polysaccharide on intestinal microflora in mice with ulcerative colitis

#### 3.6.1 Effect of HPS-80-H on the composition of intestinal flora in mice

The dysbiosis of intestinal microbiota is intricately associated with the onset and progression of UC (35). To elucidate the impact of HPS-80-H on the gut microbiota of mice afflicted with UC, we conducted an analysis of the intestinal microbial composition utilizing high-throughput sequencing targeting the 16S rRNA V3-V4 region. In the Operational Taxonomic Unit (OTU) cluster analysis, microbial diversity was assessed through the construction of a rarefaction curve. This curve plateaued with increased sequencing depth, suggesting that the majority of the microbial diversity within all samples was adequately captured (**Figure 5A**).

**Figure 5:**
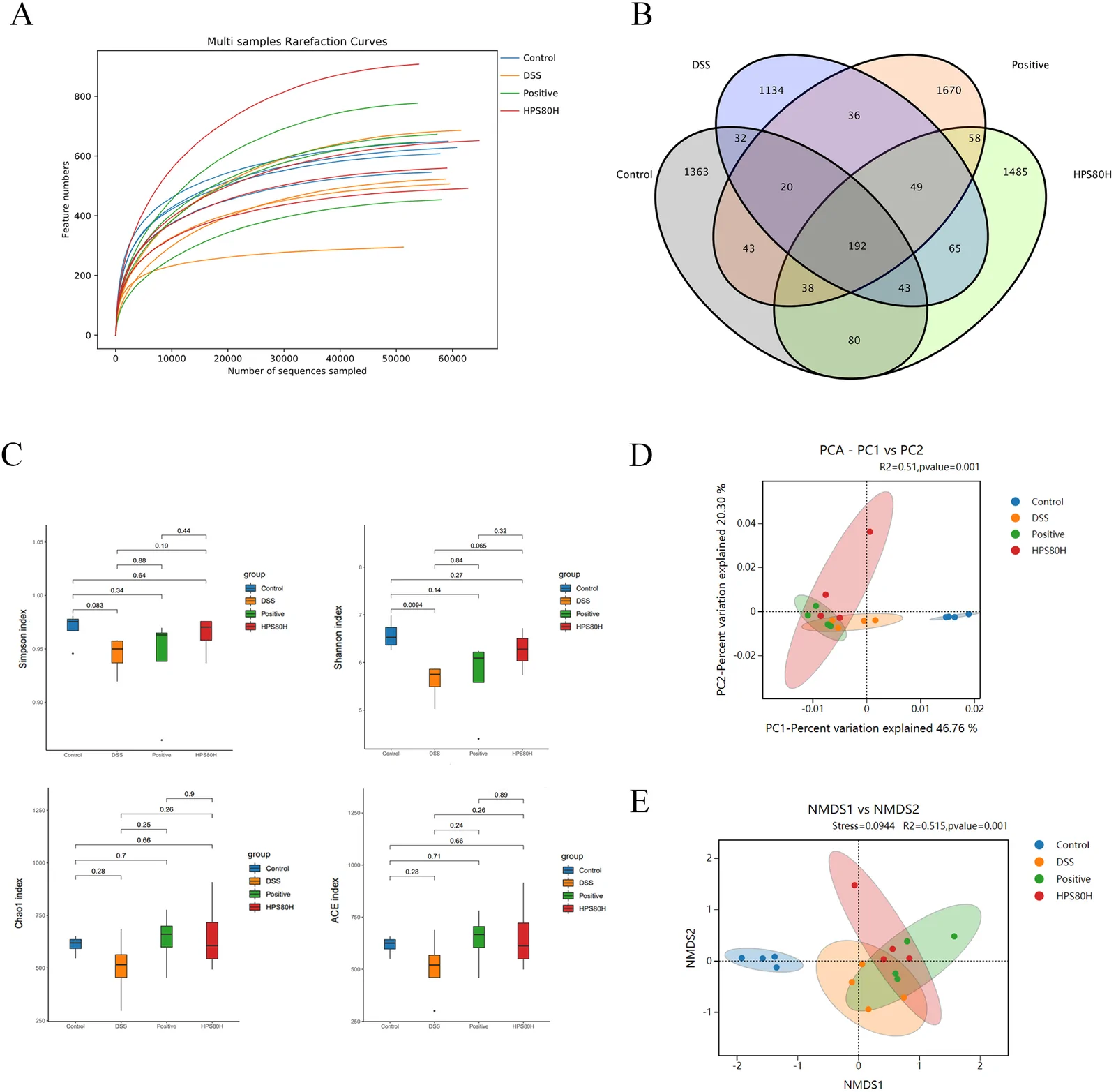
Effects of HPS-80-H on intestinal microorganisms in mice. **(A)** Rarefaction Curve. **(B)** Venn diagram of four OTUs. **(C)** Alpha diversity. (Simpson index, Shannon index, Chao1 index, ACE index.). **(D)** Principal Component Analysis (PCA). **(E)** The NMDS analysis diagram of bray-curits distance based on OTU (NMDS).

Principal component analysis (PCA) and Non-MetricMulti-Dimensional Scaling (NMDS) can reveal the β diversity analysis of intestinal microorganisms in different groups, and the similarity of the community composition of the samples can be observed by PCA and NMDS. Examination of OTU overlap among groups revealed 192 OTUs common to all groups, with 1363, 1134, 1670, and 1485 OTUs identified in the Control, DSS, Positive, and HPS-80-H groups, respectively (**Figure 5B**). In the α-diversity analysis, indices such as Chao1, Simpson, ACE, and Shannon indicated a significant reduction in community richness in the DSS group compared to the Control group, whereas HPS-80-H administration markedly enhanced community richness (**Figure 5C**).

PCA and NMDS analyses separated microbial communities into four distinct clusters (**Figures 5D, E**), demonstrating clear inter-group differences in gut-microbiota composition. Such pronounced community separation indicated that each experimental group harbored a unique intestinal microbial profile. The marked separation along the PC1 axis between the Control group and the DSS group demonstrated a significant difference in their microbial compositions (*P <* 0.001), with DSS treatment altering the intestinal microbiota in mice. The Positive group and the HPS-80-H group were positioned between the Control and DSS groups, yet neither was completely separated from the DSS group along the PC1 axis. This suggests that the microbial community compositions of the HPS-80-H and Positive groups were not identical to that of the DSS group. These findings suggest that HPS-80-H can modulate the structure of the intestinal microbial community in DSS-induced UC mice.

#### 3.6.2 HPS-80-H may improve UC in mice by increasing the relative abundance of beneficial bacteria

The ten species exhibiting the highest relative abundance at both the phylum and genus levels were analyzed using heat maps, cluster analysis, and species relative abundance histograms (**Figures 6A, B**). At the phylum level, HPS-80-H significantly enhanced the relative abundance of Firmicutes and Verrucomicrobiota, while DSS markedly increased the relative abundance of Bacteroidota and Proteobacteria. At the genus level, HPS-80-H significantly elevated the relative abundance of Akkermansia and the Lachnospiraceae_NK4A136_group. In contrast, both the DSS and Positive groups significantly increased the relative abundance of Turicibacter and Bacteroidota. Across both taxonomic levels, it was observed that HPS-80-H may modulate the intestinal microbiota by augmenting the relative abundance of beneficial bacteria such as Firmicutes, Verrucomicrobiota, Akkermansia, and the Lachnospiraceae_NK4A136_group, thereby contributing to theamelioration of ulcerative colitis.

**Figure 6:**
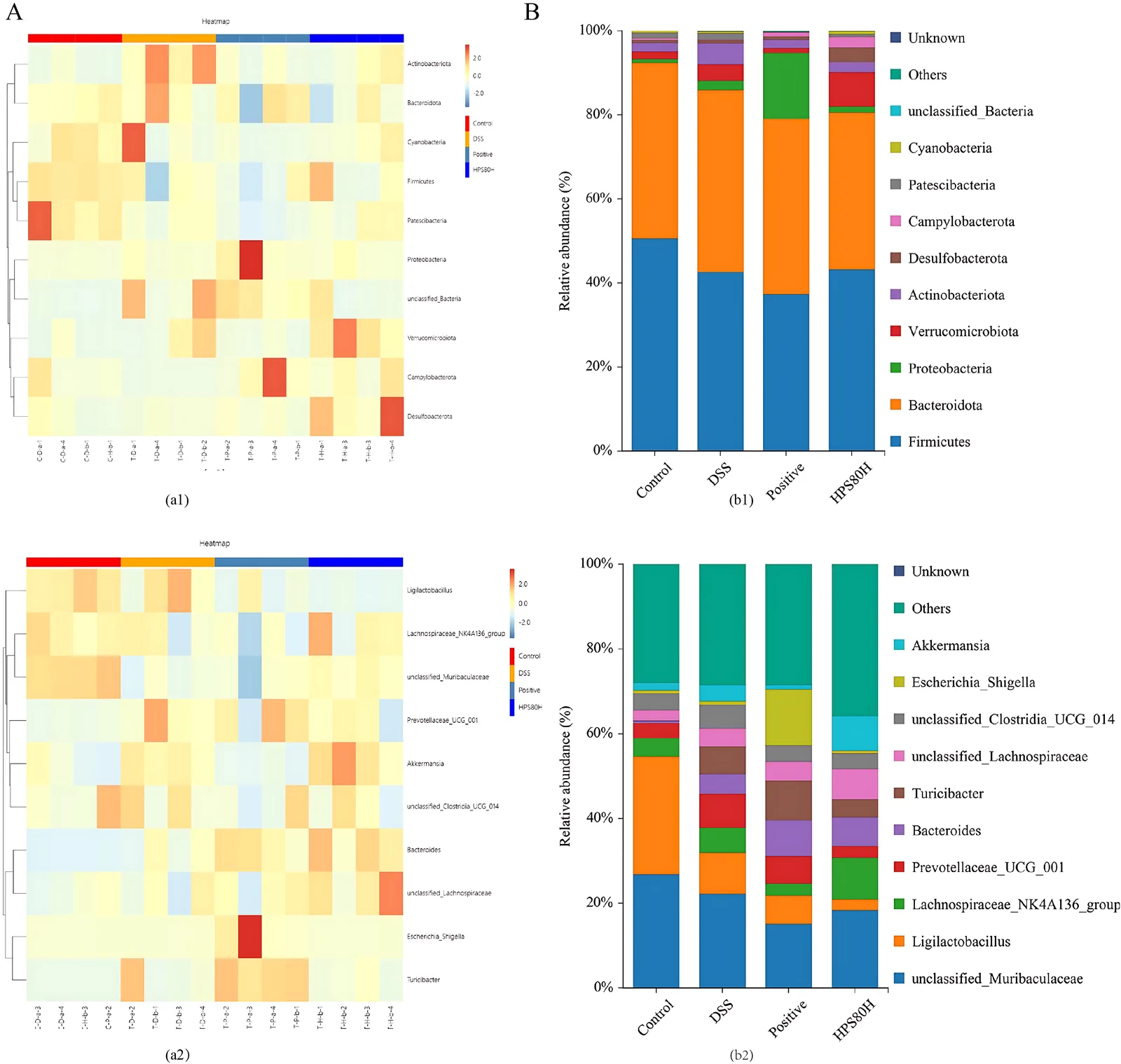
HPS-80-H causes intestinal microbial changes at the phylum and genus levels. **(A)** Heatmap (a1- at the phylum level,a2-at the genus level). **(B)** Intestinal microbial composition of the four groups of mice (b1- at the phylum level,b2-at the genus level).

In the LEfSe analysis, the Linear discriminant analysis(LDA) diagram can be examined from the taxonomic levels ranging from phylum to family. In the Control group, beneficial bacteria such as Lachnospirales (a member of the Firmicutes phylum) were predominant, whereas in the DSS group, harmful bacteria such as Coriobacteriales and Coriobacteriia were more prevalent. In contrast, beneficial bacteria like Clostridia were dominant in both the HPS-80-H group and the Positive group. The administration of HPS-80-H was found to enhance the intestinal microenvironment in mice, leading to an increased relative abundance of beneficial bacteria (**Figure 7A**). The microbial composition of the HPS-80-H and Positive groups closely resembled that of the Control group, while the microbial profile of the DSS group was markedly distinct from the other three groups. This suggests that HPS-80-H has the potential to modulate the gut flora in mice, restoring it to a more normal state (**Figure 7D**). Furthermore, compared to the DSS group, the relative abundance of Lachnospira was significantly higher in the HPS-80-H group (**Figure 7B**).

**Figure 7:**
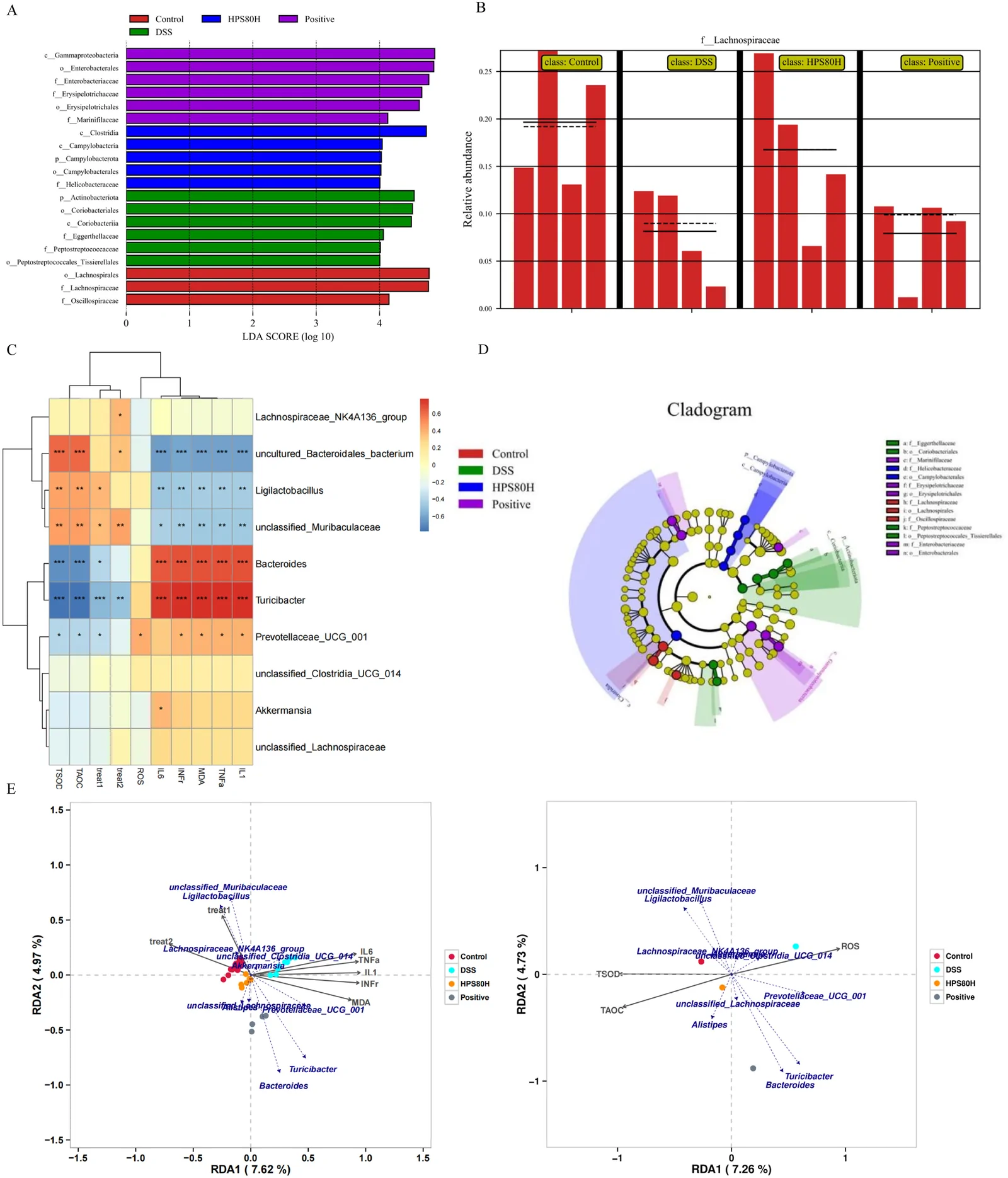
HPS-80-H treats DSS-induced UC by increasing the abundance of beneficial bacteria. **(A)** Linear discriminant analysis (LDA). **(B)** The relative abundance map of Lachnospiraceae. **(C)** Spearman correlation analysis heat map. **(D)** LEfSe analysis evolutionary branch diagram. **(E)** redundancy analysis (RDA.Treat1:Weight.Treat2:Colon length.).

#### 3.6.3 Effects of HPS-80-H on intestinal flora metabolism in mice

Utilizing Spearman correlation analysis, several microbial species demonstrated significant positive or negative correlations with colitis-related indicators, including colon length, body weight, inflammatory factors, and oxidative stress. Redundancy analysis (RDA) revealed high similarity within the four groups of samples, alongside distinct differences between the groups. The most pronounced difference was observed between the control group and the DSS group, indicating the successful establishment of the model. The HPS-80-H group was positioned between the two but closer to the control group, suggesting that HPS-80 polysaccharide exerts a notable preventive effect on intestinal flora dysbiosis in mice with ulcerative colitis (**Figure 7E**). Correlation heat map analysis further indicated that the uncultured _Bacteroidales_ bacterium, _Ligilactobacillus_, and unclassified _Muribaculaceae_ exhibited significant positive correlations with colon length and body weight, while showing significant negative correlations with inflammatory factors such as IL-6, IL-1β, IFN-γ, TNF-α, and oxidative index MDA. Conversely, Bacteroides, Turicibacter, and Prevotellaceae-UCG-001 exhibited a negative correlation with colon length and body weight, while demonstrating a positive correlation with inflammatory factors and malondialdehyde (MDA) (**Figure 7C**). Spearman correlation analysis revealed significant associations between the relative abundance of specific intestinal microbes and colitis-related phenotypic indicators. The abundances of uncultured Bacteroidales bacterium and Ligilactobacillus were positively correlated with improved colon tissue status, while Bacteroides, Turicibacter and Prevotellaceae-UCG-001 showed negative correlation with colon protective phenotypes. This modulation may contribute to the scavenging of free radicals, thereby reducing inflammatory factors and lipid peroxidation, and enhancing the body’s antioxidant capacity to achieve preventive effects.

These findings suggest that HPS-80 may ameliorate the dysbiosis of intestinal flora in UC mice by modulating the metabolic activities of the intestinal microbiota.

#### 3.6.4 The preventive effect of mesalazine enteric-coated tablets on UC in mice was not obvious

In the course of the animal experiment, mice in the Positive group were the first to exhibit symptoms of UC, including significant weight loss and hematochezia, while undergoing drug treatment, in comparison to other groups. Analysis of the species distribution histogram revealed a marked increase in the relative abundance of pathogenic bacteria such as Bacteroidota, Turicibacter, and Proteobacteria at both the phylum and genus levels in the Positive group. These characteristics closely resembled those observed in the DSS group, contrasting significantly with the Control group (**Figure 7B**). Furthermore, this pattern was more pronounced in the relative abundance profile of the genus Lachnospira, where the preventive effect of HPS-80-H aligned more closely with the Control group, whereas the Positive and DSS groups exhibited greater similarity (**Figure 7B**). Previous studies have indicated that although 5-ASA does not reverse the DSS-induced increase in the relative abundance of Bacteroidota and Proteobacteria, it can enhance the abundance of certain beneficial bacteria (36). Despite the limited therapeutic efficacy of 5-ASA in DSS-treated mice, the relative abundance of unclassified Lachnospira was noted.

This study has delineated the overall effects of HPS-80 on regulating inflammatory factors, oxidative stress, and intestinal microbiota. Building on this foundation, subsequent research will focus on in-depth exploration of its underlying molecular pathways—specifically, the upstream signaling mechanisms governing the expression of tight junction proteins (e.g., ZO-1 and occludin) will be clearly elucidated. Furthermore, while the current investigation has completed the species abundance analysis of intestinal microbiota via 16S rRNA sequencing, metabolomic data will be supplemented in the next phase to improve the research system. This will ultimately enable the definitive identification of key metabolites generated through HPS-80-mediated modulation of gut microbiota, as well as their direct functional impacts on UC.

## 4 Conclusion

In this study, 5-ASA was used as the positive control agent. Multi-index comprehensive measurements confirmed that HPS-80 confers overall protective effects against DSS-induced ulcerative colitis in mice. Collectively, our multiple animal-experiment phenotypic readouts demonstrate that HPS-80 alleviates DSS-triggered colonic injury. It acts by suppressing pro-inflammatory cytokine levels, restoring intestinal antioxidant capacity, upregulating tight-junction proteins, and remodeling gut-microbiota profiles. We hypothesize that HPS-80 protects colon tissue by dampening inflammatory responses and preserving redox homeostasis. However, observed gut-microbiota alterations are merely correlated with colitis improvement. Our present dataset cannot determine whether microbiota remodeling directly mediates HPS-80-conferred protective effects. Notably, 16S rRNA high-throughput sequencing was only performed for the high-dose HPS-80-H group. The observed microbiota-regulating features are specific to high-dose intervention and cannot be generalized to the low- and medium-dose groups without corresponding sequencing datasets. Additional sequencing for HPS-80-L and HPS-80-M will be carried out in follow-up research to systematically evaluate dose-dependent differences in gut-microbiota modulation.

This work has several notable limitations. First, we quantified only four circulating pro-inflammatory cytokines (TNF-α, IL-6, IL-1β, IFN-γ) to evaluate inflammatory status. Serum cytokine concentrations reflect systemic inflammation and may not faithfully mirror the local immune microenvironment inside colonic tissue. Moreover, anti-inflammatory cytokines (IL-4, IL-10) and Th17-associated mediators (IL-17) were not detected, which limits our ability to comprehensively assess the balance between pro- and anti-inflammatory immune responses. In addition, key proteins of the NF-κB signaling pathway were not examined, leaving us without direct evidence explaining the upstream regulatory cascade governing these pro-inflammatory mediators; only three mice from each group were subjected to IHC detection in the present study, and this small sample size may partly weaken statistical robustness for quantitative tissue-protein measurements. Second, other core signaling axes including NF-κB, the NLRP3 inflammasome, Nrf2/HO-1 and TLR4/MyD88 remained uncharacterized. *In-vitro* functional cell assays, fecal metabolomic profiling, fecal microbiota transplantation (FMT), and germ-free mouse validation experiments were also absent. Consequently, we cannot confirm a causal relationship between gut microbiota and the therapeutic efficacy of HPS-80. Direct molecular evidence supporting our proposed mechanisms is therefore incomplete, and it remains unproven whether gut microbiota serves as a critical mediator for the colon-protective effects of HPS-80. Third, endotoxin quantification was not performed for the HPS-80 polysaccharide preparation in this study. Potential immunostimulatory effects originating from endotoxin contamination cannot be fully excluded, which may interfere with the interpretation of its immunomodulatory and anti-colitis bioactivities. Further systematic studies incorporating cellular models, core signaling-pathway detection, fecal metabolomics, causal validation of microbiota, as well as endotoxin assessment for polysaccharide samples are required to fully decipher the precise molecular mechanisms underlying HPS-80-mediated alleviation of ulcerative colitis, wherein larger animal sample sizes will be adopted in further mechanistic studies to improve the reliability of quantitative tissue-protein results. In addition, the present study only verified the therapeutic efficacy and preliminary mechanism of HPS-80 in ulcerative colitis, while systematic toxicological evaluation and pharmacokinetic analysis remain unperformed. The *in vivo* safety profile, dose-toxicity relationship, and metabolic characteristics of HPS-80 require further investigation in future studies to support its translational application. Despite these limitations, our multi-phenotypic dataset provides a solid preliminary foundation for future mechanistic investigations.

With respect to gut-microbiota regulation, HPS-80-H showed more favorable trends in restoring microbial community composition compared with mesalazine, although formal statistical comparisons between these two treatments were not performed.

Accumulating evidence indicates that a broad range of natural plant-derived polysaccharides exert protective effects in DSS-induced ulcerative colitis mouse models. For example, polysaccharides from Tremella fuciformis alleviate colonic injury by regulating Foxp3^+^ T-cell responses, reshaping gut microbiota and microbial metabolites, and suppressing pro-inflammatory cytokine production (37). Polysaccharides from ‘Nanguo’ pear can mitigate intestinal oxidative stress, up-regulate tight-junction proteins such as ZO-1 and Occludin, boost short-chain-fatty-acid production, enrich beneficial taxa including Akkermansia, and preserve intestinal mucosal-barrier integrity to confer anti-colitis activity (38). Consistent with these reported polysaccharides, HPS-80 derived from Radix Hedysari exhibits multiple bio-functions, including anti-inflammatory and anti-oxidant activities, enhancement of tight-junction-protein expression, and gut-microbiota modulation. HPS-80 merits further exploration as a candidate agent for the prevention and early-stage intervention of ulcerative colitis, which differentiates it from many other polysaccharide studies that focus primarily on therapeutic treatment. In the present DSS-evoked colitis mouse model, HPS-80 rescued DSS-triggered body-weight loss and colon shortening, attenuated histopathological lesions, enriched beneficial bacterial taxa such as Firmicutes and Akkermansia, and inhibited pro-inflammatory-cytokine release.

## Data Availability

The data presented in the study are deposited in the NCBI SRA repository, accession number PRJNA1515476.

## Supplementary material

Supplementary Table 1 Changes in organ index of mice in each group (x ± s).

Supplementary Table 2 Effects of HPS-80 on the levels of TNF-α, IL-6, IL-1β and IFN-γ in serum of UC mice (x ± S). Compared with the Control group, a < 0.05, b < 0.001; compared with the DSS group, c < 0.05, d < 0.001.

Supplementary Table 3 Effect of HPS-80 on the anti-oxidation index of UC in mice. Compared with the Control group, a < 0.05, b < 0.001; compared with the DSS group, c < 0.05, d < 0.001.

Supplementary Table 4 Colonic histopathological score.
