# Supplementary material for: Polysaccharides from Radix Hedysari exhibited preventive effects against DSS-induced ulcerative colitis in C57BL/6 mice
Source: Front Immunol. 2026 Oct 2;17:1944924. doi: 10.3389/fimmu.2026.1944924 (PMC13673562; doi:10.3389/fimmu.2026.1944924)
Supplement: Supplementary file 1 [file Table1.docx]

**Table 1 Changes in organ index of mice in each group ( x ± s )**

| Group | Visceral index(g/kg) | Spleen index(g/kg) |
| --- | --- | --- |
| C | 35.57±2.17 | 2.77±0.17 |
| M | 39.76±0.68 | 3.47±0.21 |
| P | 34.14±2.02 | 2.94±0.28 |
| HPS-80-L | 35.43±2.71 | 2.76±0.43 |
| HPS-80-M | 34.56±1.64 | 2.89±0.44 |
| HPS-80-H | 37.89±2.35 | 2.99±0.25 |
