# Supplementary material for: Polysaccharides from Radix Hedysari exhibited preventive effects against DSS-induced ulcerative colitis in C57BL/6 mice
Source: Front Immunol. 2026 Oct 2;17:1944924. doi: 10.3389/fimmu.2026.1944924 (PMC13673562; doi:10.3389/fimmu.2026.1944924)
Supplement: Supplementary file 2 [file Table2.docx]

| Group | TNF-α/(ng/L) | | IL-6/(pg/ml) | | IL-1β/（ng/L) | | INF-γ/（pg/ml） | |
| --- | --- | --- | --- | --- | --- | --- | --- | --- |
|  | `X | SD | `X | SD | `X | SD | `X | SD |
| C | 245.746^d^ | 32.180 | 103.381^d^ | 10.187 | 42.300^d^ | 3.237 | 52.621^d^ | 4.192 |
| M | 401.617^b^ | 34.451 | 249.812^b^ | 12.179 | 148.337^b^ | 11.369 | 81.670^b^ | 7.726 |
| P | 276.344^d^ | 57.833 | 117.862^d^ | 17.049 | 77.944^bd^ | 11.467 | 65.024^ad^ | 2.525 |
| HPS-80-L | 296.452^ad^ | 31.860 | 149.120^bd^ | 11.009 | 132.678^bc^ | 10.965 | 77.220^b^ | 9.707 |
| HPS-80-M | 289.017^ad^ | 12.635 | 139.127^bd^ | 10.300 | 80.782^bd^ | 6.106 | 68.696^bd^ | 6.978 |
| HPS-80-H | 272.103^d^ | 21.028 | 126.211^ad^ | 13.604 | 60.990^ad^ | 10.700 | 62.380^ad^ | 2.066 |

**Table 2 Effects of HPS-80 on the levels of TNF-α, IL-6, IL-1β and IFN-γ in serum of UC mice (x ± S)**

Note: Compared with the Control group, a < 0.05, b < 0.001; compared with the DSS group, c < 0.05, d < 0.001.
