# Supplementary material for: Polysaccharides from Radix Hedysari exhibited preventive effects against DSS-induced ulcerative colitis in C57BL/6 mice
Source: Front Immunol. 2026 Oct 2;17:1944924. doi: 10.3389/fimmu.2026.1944924 (PMC13673562; doi:10.3389/fimmu.2026.1944924)
Supplement: Supplementary file 3 [file Table3.docx]

**Table 3 Effect of HPS-80 on the anti-oxidation index of UC in mice**

| Group | T-SOD/(U/mgprot) | | ROS/(U/mgprot) | | T-AOC/(U/mgprot) | | MDA/(nmol/mgprot) | |
| --- | --- | --- | --- | --- | --- | --- | --- | --- |
|  | `X | SD | `X | SD | `X | SD | `X | SD |
| C | 200.060^d^ | 25.080 | 74.210^d^ | 9.460 | 6.271^d^ | 0.603 | 13.895^d^ | 1.452 |
| M | 143.390^b^ | 18.150 | 124.000^b^ | 3.220 | 4.572^b^ | 0.332 | 20.428^bd^ | 1.209 |
| P | 170.940^ac^ | 17.320 | 87.710^ad^ | 9.520 | 6.007^d^ | 0.334 | 15.326^d^ | 1.484 |
| HPS-80-L | 149.090^b^ | 13.390 | 77.140^d^ | 6.600 | 5.083^b^ | 0.436 | 17.081^bd^ | 1.008 |
| HPS-80-M | 161.360^a^ | 23.220 | 79.960^d^ | 10.570 | 5.373^ac^ | 0.692 | 16.371^ad^ | 1.398 |
| HPS-80-H | 176.840^ac^ | 18.610 | 69.810^d^ | 7.380 | 6.085^d^ | 0.571 | 14.863^d^ | 1.293 |

Note: Compared with the Control group, a < 0.05, b < 0.001; compared with the DSS group, c < 0.05, d < 0.001.
