# Supplementary material for: Polysaccharides from Radix Hedysari exhibited preventive effects against DSS-induced ulcerative colitis in C57BL/6 mice
Source: Front Immunol. 2026 Oct 2;17:1944924. doi: 10.3389/fimmu.2026.1944924 (PMC13673562; doi:10.3389/fimmu.2026.1944924)
Supplement: Supplementary file 4 [file Table4.docx]

**Table 4 Colonic histopathological score**

| Group |  | Score |
| --- | --- | --- |
| C |  | 0 |
| M |  | 15 |
| P |  | 9 |
| HPS-80-L |  | 11 |
| HPS-80-M |  | 12 |
| HPS-80-H |  | 6 |
